# Comparison of two commercial broth microdilution panels for multidrug-resistant Gram-negative bacteria: Thermo Scientific™ Sensititre DKMGN vs. Beckman Coulter MicroScan NMDRM1

**DOI:** 10.3389/fmicb.2024.1480687

**Published:** 2024-10-16

**Authors:** Antoine Aupaix, Kamila Lamraoui, Hector Rodriguez-Villalobos, Ahalieyah Anantharajah, Alexia Verroken

**Affiliations:** Microbiology Department, Cliniques universitaires Saint-Luc, Brussels, Belgium

**Keywords:** broth microdilution, Carbapenem resistance, Gram-negative bacteria, multidrug-resistant, comparison of methods

## Abstract

**Introduction:**

Antimicrobial susceptibility testing (AST) using broth microdilution (BMD) is usually the reference method to obtain accurate minimum inhibitory concentrations and optimally manage infections with resistant organisms. Several commercial dry BMD are available for AST in clinical laboratories.

**Materials and methods:**

Two commercial BMD panels for testing of multidrug-resistant Gram-negative bacteria were compared: the Thermo Scientific™ Sensititre DKMGN and the Beckman Coulter NMDRM1, for 17 antimicrobial agents.

**Results:**

A total of 207 isolates were tested: three ATCC strains and one NCTC strain, six quality control strains from the Belgian National Antimicrobial Committee, and 197 clinical isolates, including carbapenem-resistant *Enterobacterales*, *Pseudomonas aeruginosa*, and *Acinetobacter baumannii*. The European Committee on Antimicrobial Susceptibility Testing (EUCAST) 2023 breakpoints version 13.1 were used to assign susceptibility categories.

**Discussion:**

Overall, the categorical agreement (CA) and essential agreement (EA) were both above 90%, but several useful antibiotics for the treatment of multi-resistant organisms showed CA and EA under 90%, that is, meropenem, imipenem, and colistin for *Enterobacterales* and *meropenem* and colistin for *P. aeruginosa*. For *Enterobacterales*, the NMDRM1 panel showed a significantly higher resistance rate for meropenem, imipenem, amikacin, and colistin. For carbapenems, the minimal inhibitory concentrations (MICs) were underestimated by the DKMGN panel, as already pointed out by a warning on the EUCAST website. To better assess carbapenem susceptibility in carbapenem-resistant organisms, the DKMGN panel now requires the use of a higher inoculum in the insert kit. However, for a given isolate whose susceptibility to carbapenems is not known, there is a risk of underestimating the MIC values. Our results show that colistin testing remains a challenge, highlighting the urgent need for the development of more accurate commercial methods. The use of a single commercial method cannot guarantee good precision in the determination of the MIC value for colistin.

## 1 Introduction

Antimicrobial resistance (AMR) of Gram-negative bacilli is a growing problem in medicine. The global increase in multidrug-resistant (MDR), extensively drug-resistant, and pandrug-resistant bacteria has made the prospect of reaching a therapeutic dead end with infected patients a significant concern. In particular, the treatment of MDR *Enterobacterales*, *Pseudomonas aeruginosa*, and *Acinetobacter baumannii* infections presents a major challenge. These “priority” pathogens are one of the greatest threats to human health and new antibiotics are urgently needed (de Oliveira et al., 2020; Pendleton et al., 2013). Treatment options are limited, often requiring a combination of antibiotics that increase damage to the human microbiota and have more side effects.

It is well established that antibiotic resistance is associated with a higher mortality rate, as directly illustrated by the 1.27 million human deaths in 2019 directly attributable to AMR (Antimicrobial Resistance Collaborators, 2022). The microbiology laboratory is a key player in the detection, control, and treatment of these resistant bacteria as well as in the data collection necessary for a global understanding of this thorny problem.

To optimally manage infections with MDR organisms, antimicrobial susceptibility testing (AST) results with reliable minimum inhibitory concentration (MIC) values are mandatory. Broth microdilution appears to be the ideal method and is, for certain molecules such as colistin, the only validated method (Matuschek et al., 2018). Commercial dry microdilution panels are a convenient option for performing AST in clinical laboratories. In this study, we aimed to compare two of the main panels currently on the market: the Thermo Fisher Scientific™ Sensititre DKMGN (Thermo Fisher Scientific, Waltham, MA, USA) and the Beckman Coulter MicroScan NMDRM1 (Beckman Coulter, Brea, CA, USA) for AST of *Enterobacterales*, *P. aeruginosa*, and *A. baumannii* isolates with resistance to at least one carbapenem antibiotic.

## 2 Materials and methods

The study was conducted in the microbiology laboratory of the Cliniques Universitaires Saint-Luc—UCL, a 960-bed tertiary hospital in Brussels, Belgium.

### 2.1 Reference strains and clinical isolates

In total, six Gram-negative reference strains from the Belgian National Antimicrobial Committee (NAC), three Gram-negative ATCC strains, one NCTC strain, and 197 Gram-negative clinical isolates comprising 146 *Enterobacterales* (*70 Klebsiella* spp.*, 40 Enterobacter* spp.*, 25 Escherichia coli, 8 Citrobacter* spp.*, 2 Serratia marcescens, 1 Morganella morganii*), 44 *P. aeruginosa,* and 7 *A. baumannii,* resistant to one carbapenem (excluding intrinsic resistance), were included (Table 1). Clinical isolates mainly originated from blood cultures, sputum, abdominal samples, and urine collected between January 2015 and August 2020. These isolates were recovered from a strain bank stored at minus 20°C. Duplicate isolates from the same patient or same outbreak were excluded. Identification was performed using matrix-assisted laser desorption ionization time-of-flight mass spectrometry (MALDI-TOF MS, Bruker, Bremen, Germany) with the MALDI Biotyper IVD Reference Library (version 9). AST was initially performed using the BD Phoenix® (Becton Dickinson, Franklin Lakes, NJ, USA) with the NMIC-408 or UNMIC-409 panels and/or using the disk diffusion method (AST Disks, Bio-Rad, Marnes-la-Coquette, France). All the strains were screened for carbapenemase genes using either the Coris rapid immunochromatographic test (Resist-5 OOKNV and IMP K-SeT, Coris BioConcept, Gembloux, Belgium) or the Cepheid Carba-R assay on the GeneXpert platform (Cepheid, Sunnyvale, CA, USA). If negative, the strains were tested with an in-house PCR (ISO15189 accredited) targeting *bla*_VIM_, *bla*_IMP_, *bla*_NDM_, *bla*_KPC_, and *bla*_OXA-48_ and PCR OXACARBA targeting *bla*_OXA-23_, *bla*_OXA-24_, and *bla*_OXA-58_ (Bogaerts et al., 2013). All three techniques have the ability to detect the OXA-48-like, KPC, NDM, VIM, and IMP genes/proteins. The production of OXA-23 in *Acinetobacter* species was performed using an immunochromatographic test (OXA-23K-SeT, Coris BioConcept, Gembloux, Belgium).

**Table 1:** Organisms and resistance mechanisms.

*Enterobacterales*	*N*	%	*P. aeruginosa*	*N*	%	*A. baumannii*	*N*	%	ATCC/NCTC isolates	NAC isolates
OXA-48	45	30.6	VIM	25	56.8	OXA-23	1	14.3	*E. coli* ATCC 25922	*K. pneumoniae* NDM-1 + OXA-48
NDM	33	22.4	KPC	1	2.3	OXA-24	1	14.3	*K. pneumoniae* ATCC 700603	*K. pneumoniae* NDM-1
OXA-48 + NDM	2	1.4	OXA-48	1	2.3	OXA-58	1	14.3	*P. aeruginosa* ATCC 27853	*K. pneumoniae* KPC-3
KPC	5	3.4	Other	17	38.6	NDM	1	14.3	*E. cloacae* NCTC 13406	*E. coli* OXA-48 + MCR-1
VIM	4	2.7				Other	3	42.9		*E. cloacae* VIM-1
Other	57	39.5								*A. baumannii* OXA-23
Total	146	100.0	Total	44	100.0	Total	7	100.0		

Two *Acinetobacter* isolates were sent to the National Center for Multidrug-Resistant Gram-negative Bacteria, which identified the OXA-24 and OXA-58 genes. Isolates that tested negative with PCR or immunochromatographic testing were reported as “other” in Table 1.

### 2.2 Evaluated microdilution panels

#### 2.2.1 Thermo Scientific ™Sensititre DKMGN

This panel included 17 antimicrobial agents as presented in Table 2. All tested isolates were grown on a Columbia agar plate with 5% sheep blood (Becton Dickinson, Franklin Lakes, NJ, USA), and AST was performed according to the manufacturer’s recommendations as follows: First, a 0.5 McFarland suspension of the strain was prepared in sterile water, using a nephelometer, and then we mixed 10 μL of this suspension into the Sensititre Mueller Hinton broth. The insert kit specifies that a higher inoculum of 30 μL can be used for better detection of carbapenemase-producing *Enterobacterales* (CPE) but it does not state which inoculum will give the more accurate overall MIC values. In practice, without prior knowledge of whether a clinical isolate is a CPE before performing AST, we opted to use 10 μL. We inoculated each of the 96 wells with 50 μL of the mixed suspension, using an 8-channel programmable pipette. The plate was incubated at 35 ± 1°C, and visual reading was completed after 18–24 h.

**Table 2:** Antibiotics and dilutions included in the Thermo Scientific™ Sensititre DKMGN panel.

Antibiotics	Dilutions included (mg/L)
Amoxicillin-clavulanate	4–64/2
Piperacillin-tazobactam	1–32/4
Cefotaxime	0.5–8
Ceftazidime	0.5–16
Ceftazidime-avibactam	0.5–16/4
Ceftolozane-tazobactam	0.5–32/4
Aztreonam	0.5–32
Ertapenem	0.12–2
Imipenem	0.5–16
Meropenem	0.12–16
Amikacin	4–32
Gentamycin	0.5–8
Tobramycin	1–8
Ciprofloxacin	0.06–2
Colistin	0.25–8
Tigecycline	0.25–4
Trimethoprim-sulfamethoxazole	1–8/152

#### 2.2.2 Beckman Coulter MicroScan NMDRM1

This panel included 33 antimicrobial agents as presented in Table 3. All tested isolates were grown on a Columbia Agar plate with 5% sheep blood, and AST was performed according to the manufacturer’s recommendations as follows: First, we prepared a 0.5 McFarland suspension using the Prompt Inoculation System-D (Lund and Hawkinson, 1983). This consists of an inoculation tip with a breakable collar and a bottle of diluent. We touched several bacterial colonies with the wand, which can hold a specific number of bacteria in a groove situated at the top of it. We then wiped the breakable collar to eliminate the surplus. The wand was placed in the bottle and vigorously shaken to suspend the bacteria. The suspension was poured from the bottle to a transfer lid, and the plate was then inoculated using the RENOK rehydrator/inoculator system. This is a manual pipette that simultaneously inoculates and rehydrates all 96 wells. The plate was incubated at 35 ± 1°C, and an automated reading with the MicroScan autoSCAN-4 was completed after 16–24 h.

**Table 3:** Antibiotics and dilutions included in the Beckman Coulter MicroScan NMDRM1 panel.

Antibiotics	Dilutions included (mg/L)
Ampicillin	4–8
Amoxicillin-clavulanate	8–32/2
Mecillinam	8
Ticarcillin	8–16
Piperacillin	8–16
Piperacillin-tazobactam	8–16/4
Cefazolin	16
Cefuroxime	4–8
Cefoxitin	8–16
Cefixime	1
Cefotaxime	1–32
Cefotaxime-clavulanate	0.5–4/4
Ceftazidime	1–32
Ceftazidime-clavulanate	0.25–4/4
Ceftazidime-avibactam	2–8/4
Ceftolozane-tazobactam	1–4/4
Cefepime	0.5–8
Aztreonam	1–4
Ertapenem	0.12, 0.5–1
Imipenem	1–8
Meropenem	0.12, 1–32
Amikacin	8–16
Gentamicin	2–4
Tobramycin	2–4
Norfloxacin	0.5–1
Levofloxacin	0.5–1
Ciprofloxacin	0.06, 0.25–1
Colistin	2–4
Tigecycline	1–2
Trimethoprim	2–4
Trimethoprim/Sulfamethoxazole	2–4/76
Nitrofurantoin	64
Fosfomycin	16–64

MIC was defined as the lowest concentration of the antimicrobial agent that inhibited visible growth. Each bacterial suspension used for AST was also grown on a BD Columbia plate to verify the purity of the analyzed culture. MIC results from both panels were categorized as susceptible, susceptible-increased exposure, and resistant (S/I/R) using EUCAST 2023 breakpoints v13.1, with the exception of the *Enterobacterales* tigecycline MIC, which was interpreted with EUCAST 2018 breakpoints (v8). The lowest concentration for the NMDRM1 panel is set at 1 mg/L (breakpoint is 0.5 mg/L in v13.1 and 1 mg/L in v8). In cases of discordant S/I/R results between the two microdilution panels, the NMDRM1 panel was also read manually.

### 2.3 Data analysis

#### 2.3.1 The categorical agreement (CA)

For each antimicrobial agent present in both panels defines the agreement of interpretive MIC results (S/I/R) between the two methods expressed as a percentage. The susceptibility categories are those defined by EUCAST (Turnidge and Abbott, 2022): S—susceptible, standard dosing regimen; I—susceptible, increased exposure; R—resistant.

#### 2.3.2 The essential agreement (EA)

For each antimicrobial agent present in both panels is defined as the percentage of MIC results that fall within ±1 dilution between the two panels. If the value given by one panel was outside the MIC range of the other panel, this value was reduced to the maximum or minimum value given by the panel with the narrower range. For example, if a value of 128, 64, or 32 mg/L from the first panel was compared to a value of >8 mg/L from the second panel, both values were transformed to >8 mg/L to allow direct MIC value comparison.

#### 2.3.3 Inter-rater concordance

For each antimicrobial agent, Cohen’s kappa coefficient was calculated to evaluate the level of agreement for S/I/R categorization between the two panels. We used the guidelines of Landis and Koch (1977), which interpret kappa values as follows: <0 indicates no agreement, 0–0.20 slight, 0.21–0.40 fair, 0.41–0.60 moderate, 0.61–0.80 substantial, and 0.81–1 an almost perfect agreement.

#### 2.3.4 Analysis of discrepancies

For antimicrobial agents with CA and EA under 90%, we looked for a possible link between species or resistance mechanisms and discrepancies using a 95% confidence interval on the difference in proportions. Then, to compare the MIC results of antimicrobial agents from both panels, we plotted MIC values obtained using the DKMGN panel with MIC values obtained using the NMDRM1 panel. To draw these graphics, we adjusted the MIC values as follows: ≤X was adjusted to X (for example, ≤2 mg/L became 2 mg/L), and >X was adjusted to the next dilution (for example, >8 mg/L became 16 mg/L). To evaluate whether the S/I/R categorization discrepancies were balanced between the two panels, we used McNemar’s statistical test, which accepts or rejects symmetry in discordant pairs, for each antibiotic with CA under 90%. This allowed us to assess whether one of the panels showed a significantly higher resistance rate than the other for a given antimicrobial agent. For this analysis, we reported the McNemar observed value, which is the sum of the chi-square calculated for each possible pair (S to I and I to S; S to R and R to S; and I to R and R to I). The *p*-value was interpreted with a significance threshold of 0.05. Finally, for strains showing discordant results with carbapenems, we tested the higher inoculum of 30 μL recommended by the manufacturer, to improve the detection of CPE organisms using the DKMGN panel. We reanalyzed these results in comparison with the NMDRM1 panel.

## 3 Results

### 3.1 Reference strains

A total of 10 reference strains have been included (see Table 1). Compared to the available MIC targets of ATCC/NCTC strains (The European Committee on Antimicrobial Susceptibility Testing, 2022), we measured a CA and EA of 100% with the DKMGN results. However, a discordant result in terms of CA and EA was observed with the NMDRM1 panel for the colistin MIC value of *E. coli* ATCC 25922, which showed 4 mg/L (resistant) instead of the expected value of 0.5–1. We also observed discrepencies with colistin, gentamicin and ciprofloxacin, testing ATCC and NCTC strains but no target values are available to assess these results further (Table 4).

**Table 4:** Discordant MIC results with ATCC/NCTC strains.

Antibiotics	*E. coli* ATCC 25922 MIC (mg/L)			*K. pneumonia* ATCC 700603 MIC (mg/L)		*E. cloacae* NCTC 13406 MIC (mg/L)		Discrepancies with CA or EA?
	DKMGN	NMDRM1	Target (The European Committee on Antimicrobial Susceptibility Testing, 2022)	DKMGN	NMDRM1	DKMGN	NMDRM1	
Colistin	0.5	4	0.5–1	0.5	4			CA and EA
Gentamycin				1	4			CA and EA
Ciprofloxacin						<=0.06	0.25	EA

### 3.2 EA, CA, and inter-rater concordance for clinical *Enterobacterales* isolates

Percentages of agreement and Cohen’s kappa coefficient for concordance are shown in Table 5. The majority of the tested antimicrobial agents had a CA and EA above 90%. The lowest percentages were found with meropenem, imipenem, and colistin. Concordance with Cohen’s kappa was substantial to almost perfect for the majority of the tested molecules, except for colistin and imipenem, which showed moderate concordance. Overall, the CA and EA were both above 90%.

**Table 5:** EA, CA, and Cohen’s kappa coefficient for *Enterobacterales* with NMDRM1 and DKMGN panels.

Antibiotic	*n*	Essential agreement	Categorical agreement	Kappa - correlation	Concordance
Amikacin	146	94.5% (138)	87.7% (128)	0.70	Substantial
Amoxicillin/clavulanate	146	99.3% (145)	100% (146)	1.00	Almost perfect
Aztreonam	146	97.3% (142)	92.5% (135)	0.81	Almost perfect
Cefotaxime	146	93.8% (137)	95.9% (140)	0.71	Substantial
Ceftazidime	146	89.0% (130)	94.5% (138)	0.69	Substantial
Ceftazidime/avibactam	146	97.3% (142)	98.6% (144)	0.97	Almost perfect
Ceftolozane/tazobactam	146	92.5% (135)	91.1% (133)	0.65	Substantial
Ciprofloxacin	146	95.2% (139)	93.8% (137)	0.93	Almost perfect
Colistin^1^	116	82.8% (96)	79.3% (92)	0.53	Moderate
Ertapenem	146	95.9% (140)	92.5% (135)	0.66	Substantial
Gentamycin	146	93.8% (137)	93.8% (137)	0.87	Almost perfect
Imipenem	146	69.9% (102)	66.4% (97)	0.57	Moderate
Meropenem	146	64.4% (94)	79.5% (116)	0.76	Substantial
Piperacillin/tazobactam	146	99.3% (145)	100% (146)	0.74	Substantial
Tigecycline	146	89.0% (130)	97.9% (143)	0.72	Substantial
Tobramycin	146	93.8% (137)	97.9% (143)	0.87	Almost perfect
Trimethoprim/sulfamethoxazole	146	96.6% (141)	97.9% (143)	0.97	Almost perfect
Total	2,452	90.9% (2,230)	91.9% (2,253)		

1 For the 30 *Enterobacter* spp. isolates, colistin was not tested due to an internal expert rule in the autoSCAN-4.

### 3.3 EA, CA, and inter-rater concordance for clinical *Pseudomonas aeruginosa* isolates

The CA, EA, and Cohen’s kappa results for *P. aeruginosa* are shown in Table 6. The following antimicrobial agents showed a CA or EA of under 90%: ceftazidime/avibactam, colistin, and meropenem. Concordance was moderate for meropenem, only fair for colistin, and substantial to almost perfect for all other antibiotics. Overall, the EA and CA were both above 90%.

**Table 6:** EA, CA, and Cohen’s kappa coefficient for *P. aeruginosa* with NMDRM1 and DKMGN panels.

Antibiotic	*N*	Essential agreement	Categorical agreement	Kappa - correlation	Concordance
Amikacin	44	93.2% (41)	95.5% (42)	0.90	Almost perfect
Aztreonam^1^	44	95.5% (42)	NA	NA	NA
Ceftazidime	44	95.5% (42)	93.2% (41)	0.69	Substantial
Ceftazidime/avibactam	44	84.1% (37)	95.5% (42)	0.90	Almost perfect
Ceftolozane/tazobactam	44	100% (44)	100% (44)	1.00	Almost perfect
Ciprofloxacin	44	97.7% (43)	97.7% (43)	0.91	Almost perfect
Colistin	44	86.4% (38)	86.4% (38)	0.40	Fair
Imipenem	44	93.2% (41)	95.5% (42)	0.73	Substantial
Meropenem	44	86.4% (38)	72.7% (32)	0.57	Moderate
Piperacillin/tazobactam	44	93.2% (41)	90.9% (40)	0.62	Substantial
Tobramycin	44	95.5% (42)	93.2% (41)	0.80	Substantial
Total	484	92.8% (449)	92.0% (405)		

NA, not applicable.

1 The range of MIC values proposed by the NMDRM1 panel for aztreonam (1–4 mg/L) did not allow us to calculate CA.

### 3.4 EA, CA, and inter-rater concordance for clinical *Acinetobacter baumannii* isolates

For *A. baumannii*, all antimicrobial agents showed a substantial to almost perfect concordance with Cohen’s kappa. Meropenem and tobramycin showed a CA and EA of 85.7% (6/7), and gentamicin showed a CA of 85.7% (6/7) but an EA of 100%. Overall, the EA and CA were both above 90% (Table 7).

**Table 7:** EA, CA, and Cohen’s kappa coefficient for *Acinetobacter baumannii* with NMDRM1 and DKMGN panels.

Antibiotic	*N*	Essential agreement	Categorical agreement	Kappa - correlation	Concordance
Amikacin	7	100% (7)	100% (7)	1.00	Almost perfect
Ciprofloxacin	7	100% (7)	100% (7)	1.00	Almost perfect
Gentamicin	7	100% (7)	85.7% (6)	0.70	Substantial
Imipenem	7	100% (7)	100% (7)	1.00	Almost perfect
Meropenem	7	85.7% (6)	85.7% (6)	0.84	Almost perfect
Tobramycin	7	85.7% (6)	85.7% (6)	0.70	Substantial
Trimethoprim/sulfamethoxazole	7	100% (7)	100% (7)	1.00	Almost perfect
Total	49	95.9% (47)	93.9% (46)		

### 3.5 Analysis of discrepancies

To explore discrepancies, we further analyzed AST results of antimicrobial agents with a CA and EA under 90% for *Enterobacterales* (colistin, meropenem, and imipenem) and *P. aeruginosa* (colistin and meropenem).

#### 3.5.1 Species or resistance mechanisms associated with discrepancies

Compared to all the *Enterobacterales* tested, we saw significantly more NDM-producing strains among meropenem discordant isolates (53% vs. 22%; 95% CI: 0.03–0.060) and more OXA-48-like among imipenem discordant strains (77% vs. 31%; 95% CI: 0.26–0.66). No other significant differences related to the species or resistance mechanism were observed for the other antimicrobial agents with a CA and EA under 90%, in *Enterobacterales* or *P. aeruginosa*.

#### 3.5.2 MIC value comparison

**Figure 1:**
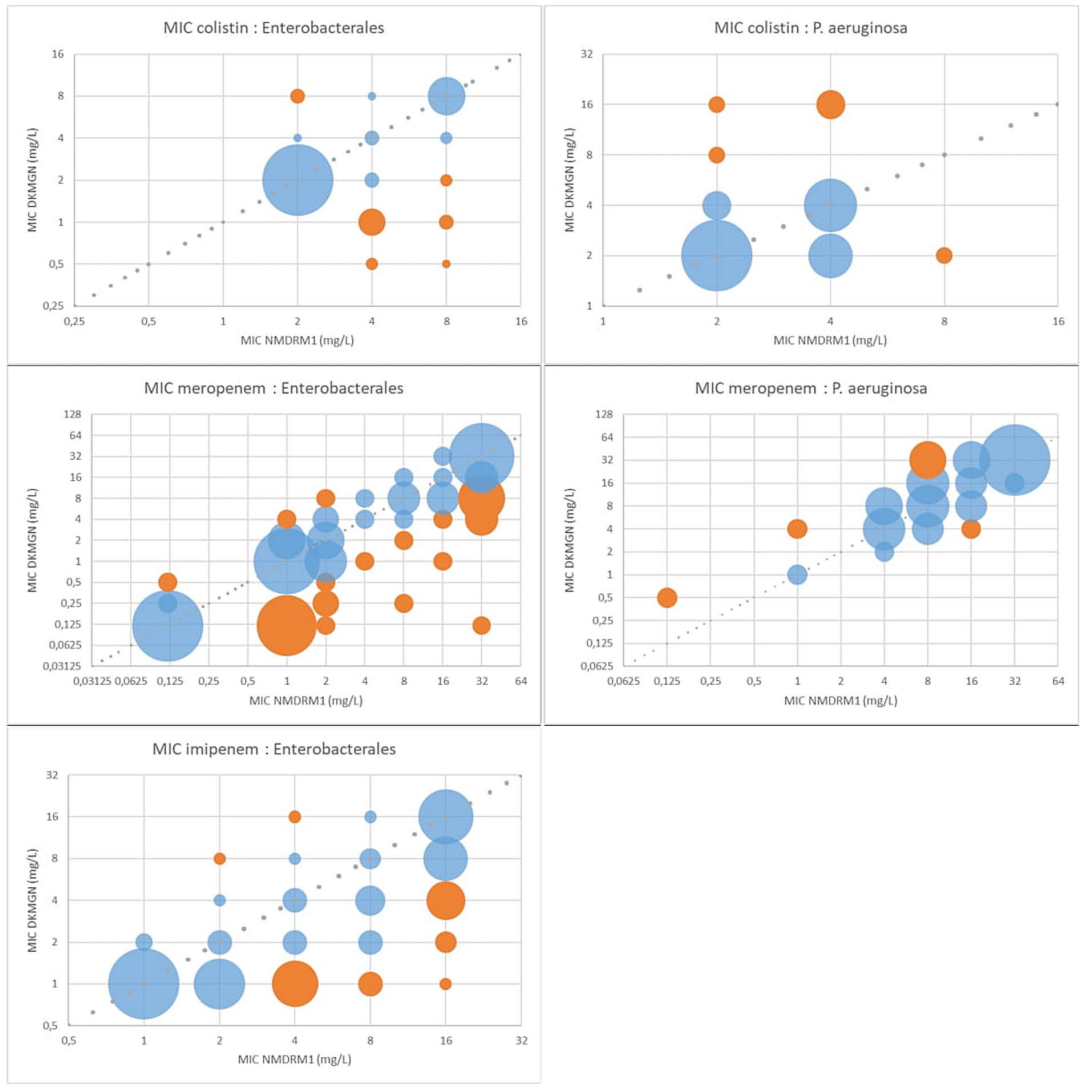
MIC value comparison between NMDRM1 and DKMGN panels for antimicrobial agents with CA and EA under 90% in *Enterobacterales* and *P. aeruginosa*. The number of isolates with the same MIC value is represented by the area of each bubble. The blue bubbles represent the MIC value within ±1 dilution between the two panels, and the orange bubbles represent the MIC discrepancies of more than one dilution.

Figure 1 shows the correlation of the MIC values for colistin, meropenem, and imipenem. Overall, *Enterobacterales* had higher MIC values using the NMDRM1 panel than the DKMGN panel for colistin and carbapenems. For *P. aeruginosa* isolates, we observed fewer discrepancies than for *Enterobacterales* and higher meropenem and colistin MIC values using the DKMGN panel.

#### 3.5.3 Discordance analysis with McNemar’s test

Concerning S/I/R discrepancies, we looked at antimicrobial agents with CA under 90%. With McNemar’s test, we could not reject symmetry in discordant pairs among *Pseudomonas* and *Acinetobacter* strains. For *Enterobacterales*, symmetry was rejected for discordant pairs with amikacin, colistin, imipenem, and meropenem. For these agents, the resistance rate was significantly higher with the NMDRM1 panel (Table 8).

**Table 8:** Discordance analysis for *Enterobacterales* and *P. aeruginosa* and *A. baumannii* using McNemar’s test.

Antimicrobial agent	McNobs	*p*-value	Symmetry	Biggest contributor
*Enterobacterales*				
Amikacin	10.9	0.00097	**Rejected**	R (NMDRM1) to S (DKMGN)
Colistin	13.5	0.00024	**Rejected**	R (NMDRM1) to S (DKMGN)
Imipenem	34.5	<0.00001	**Rejected**	I (NMDRM1) to S (DKMGN)
Meropenem	18.7	0.00031	**Rejected**	R (NMDRM1) to I (DKMGN)
Tigecycline	5.6	0.13443	Accepted	
*P. aeruginosa*				
Colistin	0.1	0.78151	Accepted	
Meropenem	1.6	0.65939	Accepted	
*A. baumannii*				
Meropenem	1	0.80125	Accepted	
Gentamycin	1	0.31731	Accepted	
Tobramycin	1	0.31731	Accepted	

McNobs, McNemar observed; R, resistant; S, susceptible; I, susceptible, increased exposure.“Rejected” was written in bold to highlight molecules with absence of symmetry.

#### 3.5.4 Results for meropenem discordant *Enterobacterales* following repeated testing of the DKMGN panel with a higher inoculum

**Figure 2:**
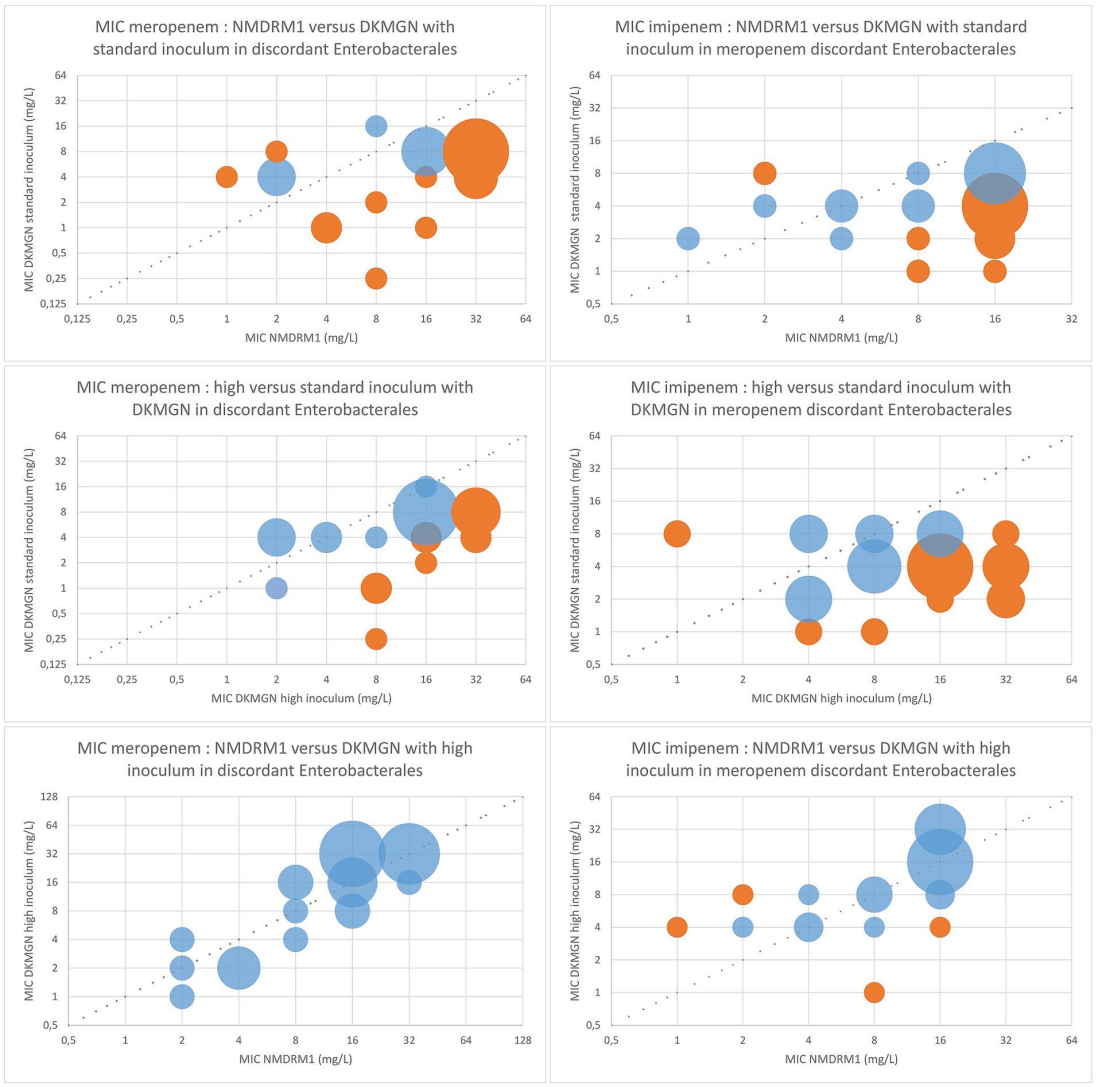
Comparison of the meropenem and imipenem MIC values between DKMGN and NMDRM1 panels with a standard and high inoculum (30 μL) for meropenem discordant *Enterobacterales*. The number of isolates with the same MIC value is represented by the area of each bubble. The blue bubbles represent the MIC values within ±1 dilution between the two panels, and the orange bubbles represent the MIC discrepancies of more than one dilution.

The 30 *Enterobacterales* isolates with no categorical agreement for meropenem were retested with the DKMGN panel with a higher inoculum of 30 μL, as recommended for the detection of CPE. Out of these 30 discordant results, CA and EA improved from 0 and 30% (standard inoculum) to 76.7 and 100% (high inoculum), respectively, compared to the NMDRM1 panel. In 24 out of 30 isolates, meropenem MICs were higher with the higher inoculum, three isolates had the same MIC value, and three isolates had a 1-fold lower dilution compared to the values obtained with the standard inoculum. Similarly, the use of a high inoculum improved the CA of imipenem from 40 to 80% and the EA from 50 to 93% compared to the NMDRM1 panel (Figure 2). Regarding meropenem MICs, 13 isolates showed an increase of >1 dilution with the higher inoculum, compared to the standard one. Among these 13 isolates, we saw significantly more NDM-producing strains than in the 30 meropenem discordant isolates (85% vs. 53%; 95% CI: 0.59–0.04). No significant difference in terms of resistance type was found for imipenem.

Considering the other antimicrobial agents, the high inoculum increased the ceftazidime/avibactam MIC values in one strain (from 2 mg/L for the standard inoculum to 8 mg/L) resulting in one more EA discordance, and increased the ertapenem MIC values in one strain (from ≤0.12 mg/L to >2 mg/L) resulting in one less discordance in CA and EA, compared to the NMDRM1 panel. No other changes in MIC were observed for any of the other antibiotics tested.

## 4 Discussion

This study aimed to compare two commercial broth microdilution panels for AST of multidrug-resistant Gram-negative bacteria: the Thermo Scientific™ Sensititre DKMGN and the Beckman Coulter MicroScan NMDRM1. Our study focused on carbapenem-resistant Gram-negative bacteria, considering that the treatment of associated infections requires accurate MIC values for broad-spectrum antibiotics, ideally obtained through microdilution.

For *Enterobacterales*, we found good overall performance with EA and/or CA above 90% for most antibiotics except for colistin, meropenem, and imipenem. We observed higher MIC values using the NMDR1 panel for these agents, which is supported by the analysis of categorical discordance using McNemar’s test. Concerning S/I/R differences, the presence of OXA-48-like and NDM in *Enterobacterales* was associated with discordances for imipenem and meropenem, respectively.

Discrepancies in commercial dry broth microdilution panels for testing carbapenem susceptibility in carbapenem-resistant organisms have already been reported (Antonelli et al., 2019). In a study by Antonelli et al., the Sensititre panel “ITGNEGF” (ITGNEGF, ThermoFisher Scientific, Massachusetts, USA) and, to a lesser extent, the MicroScan panel “Neg MIC panel type 44” (NM44, Beckman Coulter, California, USA) tended to underestimate resistance to imipenem and meropenem in KPC-producing *E. coli*, compared to a reference in-house microdilution method. To our knowledge, this study is the second to evaluate the MicroScan NMDRM1 panel. Poirel et al. (Inc MG, n.d.) previously evaluated this panel against an in-house frozen panel. Their study was carried out on 202 non-duplicate Gram-negative MDR clinical isolates, including 28.2% extended-spectrum beta-lactamase (ESBL), 52.5% carbapenem-resistant, and 26.7% colistin-resistant strains. The authors found an excellent correlation with frozen panels used as reference, with the EA ranging from 95 to 97.5% and CA ranging from 93 to 96%. The majority of categorical errors were found in *E. coli* with ertapenem and were linked to MIC values that were close to the breakpoint. False resistance to colistin was reported in 2 *P. aeruginosa* strains. The difference in the selected strain populations in the evaluation of Poirel et al. compared to ours may explain their higher agreement data results.

Analyzing MIC distribution, we observed a high rate of colistin resistance in carbapenem-resistant *Enterobacterales* (CRE) isolates (23.9% for DKMGN and 39.3% for NMDRM1). As suggested by the results of ATCC *E. coli* 25922, NMDRM1 may be overestimating MIC values for colistin, although the resistance rate found with DKMGN also appears to be high. Interestingly, several studies (Bir et al., 2022; Capone et al., 2013; Ngbede et al., 2021; Rojas et al., 2017) report high rates of colistin resistance in CRE, ranging from 5 to 36%, especially in carbapenemase-producing *Klebsiella species*. Reports of outbreaks of clonal *K. pneumoniae* resistant to both carbapenems and colistin emphasize the importance of having accurate antibiotic susceptibility test panels available (Marchaim et al., 2011; Sharma et al., 2022; Otter et al., 2017). It is also important to note that both plates are made of polystyrene, which has been shown to give less consistent results than glass-coated plates (Singhal et al., 2018).

A warning published on the EUCAST website (EUCAST, 2018) in November 2018 concerning an issue with meropenem in the Thermo Scientific™ Sensititre DKMGN panel stated that the 10 μL recommended inoculum used in the assay may lead to lower meropenem MIC values than expected in carbapenem-resistant *E. coli* and *K. pneumoniae*. We were able to confirm that an inoculum change from 10 to 30 μL using the DKMGN panel resulted in significant changes in MIC values for meropenem and imipenem testing *Enterobacterales*. This observation is highly important considering that carbapenems remain an essential therapeutic choice for the treatment of CPE-related infections. Since the EUCAST warning, the Sensititre insert kit recommends using the standard inoculum for AST of *Enterobacterales* and non-*Enterobacterales*, while suggesting a higher inoculum to help in the detection of resistance mechanisms. This approach raises a difficulty. Without knowing in advance whether a given strain is or is not carbapenem-resistant, an initial AST is made with the standard inoculum, followed 24 h later by a second AST with a higher inoculum of 30 μL, to confirm carbapenem MICs. This approach doubles the cost in terms of reagents and turnaround time. Another approach would be to directly use the higher inoculum for all tested isolates, but it would require a whole new validation for all measured antibiotics. This issue may be partially explained by the inoculum effect. Although the EUCAST and CLSI guidelines (Clinical and Laboratory Standards Institute, 2015; EUCAST, 2018; eucast: MIC determination, n.d.), following the recommendations of the International Standards Organization (ISO 20776-1), accept a variation in the inoculum tested for AST (5 × 10^5^ CFU mL^−1^ with an acceptable range of 2 × 10^5^–8 × 10^5^ CFU mL^−1^), the use of a more precise inoculum may be required to correctly assess CRO susceptibility. Other studies have shown that small variations in inoculum can have a significant impact on meropenem susceptibility testing (Smith and Kirby, 2018; Ogawa et al., 2019; Danjean et al., 2022). Interestingly, in our study, strains that showed significant changes (>1 dilution) in meropenem MIC values with the higher inoculum were almost exclusively NDM-producing isolates. The meropenem MIC results found using the DKMGN panel with the higher inoculum were closer to the published meropenem MIC values of NDM-producing *Enterobacterales* (Fattouh et al., 2016). This is supported by a recent study by Golikova et al. (2023) that studied the meropenem inoculum effect in non-carbapenemase-producing, OXA-48-like, KPC-, and NDM-producing *K. pneumoniae*. The authors found the highest inoculum-related MIC shifts in NDM-producing isolates compared to other carbapenemase- and non-carbapenemase-producing isolates.

For *P. aeruginosa* isolates, the overall CA and EA were both above 90%. However, two antimicrobial agents—colistin and meropenem—showed CA and EA values under 90%. MIC plots for these antibiotics appeared to show higher values using the DKMGN panel. Ceftazidime-avibactam showed an EA under 90% but a good *CA.* A study by Ito et al. reported satisfactory performance when testing ceftazidime–avibactam using the NMDRM1 panel, with a CA of 98.6% and an EA of 100% compared to a reference BMD test (Ito et al., 2021). Regarding colistin, the majority of isolates had a MIC value close to the breakpoint. The recent change in the colistin susceptibility breakpoint in EUCAST v13.1, from 2 to 4 mg/L, significantly impacted the CA, increasing it from 70.5% (with the previous breakpoint of 2 mg/L) to 86.4% (with the current breakpoint of 4 mg/L). As mentioned above, one study already found good performance with *P. aeruginosa* strains for all antimicrobial agents of the NMDRM1 panels, including colistin (Inc MG, n.d.). However, colistin discrepancies with another MicroScan panel (NM44) have been previously reported in a study conducted on non-fermenting bacteria (*P. aeruginosa*, *A. baumannii*, and *S. maltophilia*), where the authors found a CA of 64.1% compared to a reference broth microdilution (BMD) panel (Jayol et al., 2018). As noted in other studies, MIC determination for colistin comes with methodological difficulties and, particularly for *P. aeruginosa* isolates, we could expect categorical errors even with the best-calibrated method, as the EUCAST susceptibility breakpoint is set at 2 mg/L, whereas the ECOFF value is 4 mg/L^4^. The new breakpoint in v13.1 therefore appears to be more convenient. Once again, the polystyrene contained in the plate could also impact the colistin results. Finally, concerning meropenem, we saw a majority of isolates (55% with the DKMGN panel and 66% with the NMDRM1 panel) with MIC values around the resistance cut-off (between 4 and 16 mg/L). This could explain the low CA value and the better EA.

For *A. baumannii*, the overall CA and EA were both above 90%. Three antibiotics (gentamicin, tobramycin, and meropenem) had CA and/or EA values under 90%; however, all discordant results were linked to a single isolate. The low number of *Acinetobacter* tested (*N* = 7) does not allow us to draw any conclusions. Concerning the Sensititre DKMGN panel for *A. baumannii* isolates, one publication found a good CA (99.1%) compared to a reference BMD method but a low EA of 60.6%. These discrepancies resulted from higher MIC values using the DKMGN panel, but the clinical implication was uncertain because the S/I/R categorization was identical between panels (Chung et al., 2022).

Finally, we note that the NMDRM1 panel offers a broader choice of antimicrobial agents than the DKMGN panel. The additional testing for nitrofurantoin and fosfomycin may be useful in the subsequent treatment of CRE urinary tract infections. Conversely, for some molecules, the MIC value ranges are limited on the NMDRM1 panel. For example, only concentrations of 1 and 2 mg/L are tested for tigecycline, which does not allow interpretation of *E. coli* isolates with the recent EUCAST criteria (breakpoint set at 0.5 mg/L). We further regret the absence of temocillin in both panels, an antibiotic that is not only useful for the treatment of ESBL-producing *Enterobacterales* but also important for the detection of OXA-48-like carbapenemase.

Our study has some limitations. First of all, this is a comparison of two commercial dry broth microdilution methods. In the absence of a reference method, we cannot know which panel is more accurate. With regard to colistin resistance, the concerned strains were not systematically sent to the national reference center for confirmation and typing (in particular for the MCR-1 gene). Regarding IMP isolates, our detection methods did not detect all subtypes. Finally, there are methodological differences between the two panels: The reading process was executed visually for the DKMGN panel and automatically using the MicroScan autoSCAN-4 for the NMDRM1 panel. Nevertheless, for all discordant results, the NMDRM1 panels were also read visually, and no difference was found compared to the autoSCAN-4 results. Preparation of the 0.5 McFarland inoculum also differs between the methods: the DKMGN panel requires a nephelometer while for the NMDRM1 panel, the inoculum is directly prepared by the Prompt Inoculation System-D. The latter has, however, been validated (Lund and Hawkinson, 1983).

## 5 Conclusion

The Thermo Scientific™ Sensititre DKMGN and the Beckman Coulter NMDRM1 dry broth microdilution panels are simple tools for the AST of MDR organisms in routine laboratories. However, the results of some antibiotics, namely, carbapenems and colistin in *Enterobacterales*, and colistin and meropenem in *P. aeruginosa*, can differ significantly between the two panels, depending on the type of carbapenemase gene produced by the organism. We confirmed the underestimation of the MIC values for carbapenems using the DKMGN panel, which required a higher inoculum as pointed out by the EUCAST recommendations. Our results showed that colistin testing remains a challenge, highlighting the urgent need for the development of more accurate commercial methods. The use of a single commercial method cannot guarantee good precision in the determination of the MIC value for colistin. A second method should be used when this antibiotic is the last therapeutic option.

## Data Availability

The raw data supporting the conclusions of this article will be made available by the authors, without undue reservation.
